# Kinetics of Nitrous Oxide (N_2_O)-reducing Activity of *Bradyrhizobium ottawaense* by an Automated Analysis

**DOI:** 10.1264/jsme2.ME25070

**Published:** 2026-01-28

**Authors:** Manabu Itakura, Kiwamu Minamisawa

**Affiliations:** 1 Graduate School of Life Sciences, Tohoku University, Sendai 980-8577, Japan

**Keywords:** bradyrhizobia, *nosZ*, kinetics, nitrous oxide, N_2_O reduction

## Abstract

The biological reduction of N_2_O, a potent greenhouse gas, is crucial for environmental sustainability. We developed an automated system for continuous N_2_O monitoring in the gas phase of a flask containing an anaerobic bradyrhizobial culture, and then exami­ned the kinetic parameters of bacterial N_2_O reduction. The maximum reaction rate (*V*_max_) was approximately 61-fold higher for *Bradyrhizobium ottawaense* SG09 (1,471‍ ‍nmol h^–1^ 10^9^ cells^–1^) than for *B. diazoefficiens* USDA110 (24‍ ‍nmol h^–1^ 10^9^ cells^–1^). Our kinetics anal­ysis confirmed that SG09 maintained higher N_2_O-reducing activity than USDA110 even at the atmospheric concentration of N_2_O (0.34 ppm).

Atmospheric nitrous oxide (N_2_O) is a major greenhouse gas that contributes to both global warming and ozone layer depletion (Ravishankara *et al.*, 2009; IPCC, 2021). With a global warming potential that is approximately 300-fold that of carbon dioxide (CO_2_) and a long atmospheric lifetime, N_2_O exerts long-term effects on Earth’s radiative budget. Agricultural activities—particularly the application of nitrogen fertilizers—are the largest source of anthropogenic N_2_O emissions (Tian *et al.*, 2020). Therefore, the mitigation of these emissions is an urgent challenge for establishing sustainable agriculture.

N_2_O is generated via several processes by diverse soil bacteria, fungi, and archaea from nitric oxide (NO) during incomplete denitrification as well as during nitrification (Butterbach-Bahl *et al.*, 2013). Only one bacterial enzyme, N_2_O reductase (encoded by *nosZ*), reduces N_2_O to N_2_ (Kuypers *et al.*, 2018).

Soybean fields emit N_2_O from degraded root nodules (Sánchez and Minamisawa, 2019). An inoculation with *Bradyrhizobium diazoefficiens*, carrying the clade I *nosZ* gene, was previously shown to reduce N_2_O emissions from soybean fields (Itakura *et al.*, 2013), which was subsequently confirmed in Japan (Akiyama *et al.*, 2016), France (Hénault *et al.*, 2022), and South America (Obando *et al.*, 2022). Under anaerobic free-living conditions, the N_2_O-reducing activities of *Bradyrhizobium ottawaense* were ~5-fold those of *B. diazoefficiens* USDA110, and were attributed to higher *nosZ* expression in *B. ottawaense* (Wasai-Hara *et al.*, 2023).

The ability of N_2_O-reducing rhizobia to mitigate N_2_O is crucial for sustainable agriculture. However, to maximize their effectiveness, a quantitative understanding of the reaction kinetics of N_2_O reductase is essential. A kinetic anal­ysis reveals the Michaelis constant (*K*_m_), indicating the enzyme’s affinity for N_2_O, and the maximum reaction rate (*V*_max_), showing its maximum reduction capacity. Although these parameters have been reported as important indicators in wastewater treatment and farmland, a kinetic anal­ysis of the N_2_O-reducing activity of symbiotic (brady)rhizobia has yet to be conducted (Yoon *et al.*, 2016; Suenaga *et al.*, 2018, 2019; Qi *et al.*, 2022; Wang *et al.*, 2023; Hiis *et al.*, 2024).

Conventional GC-based N_2_O measurements typically rely on a manual gas injection, hindering real-time monitoring capability. While automated systems (*e.g.*, GC or N_2_O microelectrodes) exist, their high cost or operational complexity limits broad accessibility (Molstad *et al.*, 2007; Suenaga *et al.*, 2018). To address this knowledge gap, we conducted a kinetic anal­ysis of the N_2_O-reducing activity of soybean bradyrhizobia. We developed an automated, continuous N_2_O measurement system using a gas chromatograph to evaluate the kinetics of the high N_2_O-reducing activity of *B. ottawaense* and the reference N_2_O-reducing activity of *B. diazoefficiens* (Wasai-Hara *et al.*, 2023).

The measurement system connects a sample flask to a GS5100S Auto Gas Sampler (sample loop volume 0.3‍ ‍mL; GL Sciences) and uses a peristaltic pump (AC-2110 II; ATTO Corporation) to circulate the gas phase between them (Fig. 1). The sampler is connected to a gas chromatograph (GC-2014; Shimadzu) equipped with an electron capture detector (ECD). The system automatically and continuously measures N_2_O and O_2_ concentrations in the gas phase by injecting the gas circulating in the sample loop into the GC at fixed time intervals (Fig. 1 and S1). The sampler’s cycle consists of “Charge”, “Balance”, “Injection”, and “Interval” steps, with customizable durations and cycle counts. The “Injection” time is the period of time during which the gas from the sample loop flows into the GC for measurement; the peristaltic pump continuously circulates gas from the flask through the sample loop during the other steps.

GC calibration was performed using N_2_O gas concentrations of 10, 50, 100, 500, 1,000, and 4,000 ppm prepared in N_2_ within cell-free assay flasks. The automated system yielded a linear calibration curve (*R*^2^=0.996, *n*=5) for the quantitative measurement of N_2_O concentrations throughout the experiment (Fig. S2). Since 0.3‍ ‍mL of the GC carrier gas (N_2_) was injected into the flask during each cycle, the measured N_2_O concentration (*C*_measured_) was corrected (*C*_corrected_) to eliminate this mechanical effect using the formula below.

$$C_{\text{corrected}}=C_{\text{measured}}\times (1+\frac{V_{\text{injection}}}{V_{\text{headspace}}}{)}^{n-1}$$

where *V*_injection_ is 0.3‍ ‍mL, *V*_headspace_ is the headspace volume of the sample flask, and *n* is the sampling number.

SG09 and USDA110 were cultured at 30°C for 4–6 days in 200‍ ‍mL of HM medium (Cole and Elkan, 1973). The cultures were then centrifuged at 9,000×*g* for 10‍ ‍min, and the supernatant was discarded. The pellets were resuspended in modified HMM medium (Sameshima-Saito *et al.*, 2004) containing trace metals (0.55‍ ‍μM Na_2_MoO_4_·2H_2_O, 1‍ ‍μM FeCl_2_, and 1‍ ‍μM CuSO_4_·2H_2_O) and L-arabinose (5‍ ‍g‍ ‍L^–1^) to OD_660_ of 0.5 (path length of 10‍ ‍mm, UV-1200; Shimadzu). Cell numbers were counted using a hemocytometer, and cell‍ ‍numbers per unit of turbidity were approximately 1.9×10^9^‍ ‍cells‍ ‍mL^–1^ OD_660_ for both strains.

The bacterial suspension was transferred to a 200-mL Erlenmeyer flask containing a stir bar: 25‍ ‍mL of SG09 and 100‍ ‍mL of USDA110. The flask was then connected to the N_2_O measurement system and maintained at 30°C in a water bath with constant agitation by a magnetic stirrer (estimated speed: ~1,200‍ ‍rpm) throughout the measurement period. The system was purged with N_2_ gas at 200‍ ‍mL‍ ‍min^–1^ for 15‍ ‍min to establish anaerobic conditions. Pure N_2_O gas (100%) was then added to the gas phase through the gas addition port (Fig. 1) to a final concentration of ~2,000 ppm for SG09 and ~1,000 ppm for USDA110 to allow for the observation of clear kinetic differences within the measurement period. In the SG09 kinetic anal­ysis, the process of‍ ‍adding N_2_O gas was repeated three times after N_2_O depletion. A peristaltic pump was used to circulate the gas phase between the flask and the Auto Gas Sampler at 25‍ ‍mL‍ ‍min^–1^. The sampler was set with a cycle of “Charge” for 60‍ ‍s, “Balance” for 10‍ ‍s, “Injection” for 240‍ ‍s, and “Interval” for 50‍ ‍s, allowing for the measurement of N_2_O and O_2_ concentrations every 6‍ ‍min.

By using the corrected N_2_O concentrations, we calculated the substrate concentration, [*S*], from the midpoint of two consecutive measurement points and the reaction rate, *v*, from the slope between them. The relationship between [*S*] and *v* was fit to the Michaelis–Menten equation:

$$v=\frac{V_{\text{max}}\times [S]}{K_{m}+[S]}$$

using the least squares method in R v. 4.2.2 software (R Core Team, 2022) to assess the *K*_m_ and maximum reaction rate (*V*_max_) of N_2_O-reducing activity (Suenaga *et al.*, 2018, 2019; Wang *et al.*, 2023). Cell-specific *V*_max_ was calculated using the average cell number measured before and after the kinetic anal­ysis. The kinetic anal­ysis was performed for each species in independent triplicate experiments.

To validate the N_2_O measurement system, we connected a flask containing 25‍ ‍mL of HM medium without bacteria. After purging the flask with N_2_, ~1,400 ppm of N_2_O gas was‍ ‍added. N_2_O and O_2_ concentrations were measured at 6-‍min intervals for 6 h. The N_2_O concentration gradually decreased with each measurement (Fig. S3). We attributed this to the introduction of 0.3‍ ‍mL of the GC carrier gas (N_2_) into the flask per measurement cycle via the sample loop. When the N_2_O concentration was corrected for this influx using the formula defined above, the decrease in the N_2_O concentration was almost eliminated (Fig. S3). On the other hand, the O_2_ concentration was below the detection limit (0.05%) after N_2_ purging and remained very low (<0.1%) thereafter, confirming that anaerobic conditions were maintained.

We analyzed the N_2_O-reducing activities of SG09 and USDA110 using the N_2_O measurement system. After N_2_O was added to the gas phase of the SG09 culture at ~2,000‍ ‍ppm, the N_2_O concentration rapidly decreased to below the detection limit (<5 ppm) in approximately 60‍ ‍min (Fig. 2A). Subsequent additions of N_2_O resulted in similar decreases (Fig. 2A). In contrast, when USDA110 was measured at the same cell density as SG09, the addition of N_2_O at 1,000 ppm decreased to only ~600 ppm after 720‍ ‍min (Fig. S4). To ensure sufficient kinetics data while avoiding potential density-dependent effects, we maintained cell density, but used 100‍ ‍mL of a bacterial suspension for the USDA110 anal­ysis. Nevertheless, USDA110 took 200‍ ‍min to reduce N_2_O below the detection limit (Fig. 2B).

After the correction of measured N_2_O concentration data, we calculated [*S*] and *v* and fit the data to the Michaelis–Menten equation using the least squares method to assess *K*_m_ and *V*_max_ (Fig. 2C and D). The *K*_m_ of SG09 was 227.7‍ ‍ppm and *V*_max_ was 1,470.9‍ ‍nmol h^–1^ 10^9^‍ ‍cells^–1^. The *K*_m_ of USDA110 was 68.1 ppm and *V*_max_ was 23.6‍ ‍nmol h^–1^ 10^9^‍ ‍cells^–1^. The *K*_m_ and *V*_max_ of SG09 were ~3× and ~60× those of USDA110 (Table 1). At the ambient atmospheric concentration of N_2_O (0.34 ppm), the calculated N_2_O reduction rate was 2.24‍ ‍nmol h^–1^ 10^9^ cells^–1^ for SG09 and 0.12‍ ‍nmol h^–1^ 10^9^ cells^–1^ for USDA110. This suggests that SG09 maintained high N_2_O-reducing activity, even at atmospheric N_2_O (Table 1). The *K*_m_ values of both strains were similar to those of *nosZ*-carrying bacteria in clades I and II, and while the *V*_max_ of SG09 was equivalent, that of USDA110 was markedly low (Table 2).

Although N_2_O microelectrodes enable the real-time measurement of dissolved gas in wastewater (Suenaga *et al.*, 2018, 2019; Qi *et al.*, 2022; Wang *et al.*, 2023), they are expensive, fragile, and unsuitable for crucial gas-phase N_2_O measurements in soil microbial studies. While Molstad *et al.* (2007) established a high-throughput multi-sample GC system, we designed a simpler alternative using existing components (Fig. 1). Our system prioritizes a simpler operation and detailed, continuous measurements for a single sample. This simple design allows for the easy measurement of N_2_O-reducing activity by those with the ability to prepare a bacterial culture. It offers several other advantages: the nitrogen gas purge line facilitates the easy establishment of anaerobic conditions. Its flexibility in changing the gas species and composition of the purge line also enables measurements under aerobic and microaerobic conditions. The system’s modular design also allows for easy adaptation to measurements of other greenhouse gases once the appropriate GC detector and column are used. Furthermore, it is broadly applicable, allowing not only for the sampling of microbial cells for a subsequent gene expression anal­ysis, but also for N_2_O reduction measurements from a wide range of samples, including bacterial cultures, root nodules, soil, and microbial carriers.

Our kinetic anal­ysis supports previous findings showing that *B. ottawaense* SG09 exhibited higher N_2_O-reducing activity than *B. diazoefficiens* USDA110 (Wasai-Hara *et al.*, 2023). The estimated *V*_max_ of SG09 was 1,470.9‍ ‍nmol h^–1^ 10^9^‍ ‍cells^–1^—within the reported range of 1,350–3,000‍ ‍nmol h^–1^ 10^9^‍ ‍cells^–1^. In contrast, the *V*_max_ of USDA110 was 23.6‍ ‍nmol h^–1^ 10^9^ cells^–1^—markedly lower than reported values of 300–500‍ ‍nmol h^–1^ 10^9^ cells^–1^. This discrepancy may be attributed to differences in experimental conditions: the present study used cells grown under aerobic conditions, while the previous study used cells precultured overnight under N_2_O respiration conditions to fully induce N_2_O reductase activity before measurements (Wasai-Hara *et al.*, 2023). However, the N_2_O-reducing activity of USDA110 measured under non-induced conditions was ~12‍ ‍nmol h^–1^ 10^9^ cells^–1^ (Itakura *et al.*, 2008), similar to the *V*_max_ estimated herein. These results suggest that the kinetic parameters obtained using our novel automated continuous measurement system are reliable and also indicate that SG09 has an intrinsic ability to rapidly express or activate N_2_O reductase at a high level without the need for pre-induction. This characteristic gives SG09 a marked advantage for N_2_O mitigation in dynamic soil environments, where N_2_O levels fluctuate.

SG09 had a higher *K*_m_ value than USDA110. While a lower *K*_m_ value indicates a higher affinity for the substrate (N_2_O), suggesting that USDA110 is more reactive under low N_2_O concentrations, our calculations showed that the SG09 reaction rate still surpassed that of USDA110 at an atmospheric N_2_O level (0.34 ppb). Therefore, from the perspective of N_2_O removal from soils where it is being actively generated, SG09 may be regarded as superior due to its higher reaction rate even at an atmospheric concentration. This result highlights the importance of evaluating both *K*_m_ and *V*_max_ when assessing the overall N_2_O mitigation potential of a microbial strain under realistic environmental conditions.

Comparisons of the *K*_m_ and *V*_max_ values of SG09 and USDA110 with those of previously reported clades I and II *nosZ*-carrying bacteria showed that *K*_m_ values were similar. However, the *V*_max_ value of USDA110 was low, whereas that of SG09 was equivalent to that of *Cloacibacter* sp. CB-01, a strain reported to be effective in reducing farmland N_2_O emissions (Hiis *et al.*, 2024), suggesting the similar effectiveness of *B. ottawaense* SG09 (Table 2).

## Citation

Itakura, M., and Minamisawa, K. (2026) Kinetics of Nitrous Oxide (N_2_O)-reducing Activity of *Bradyrhizobium ottawaense* by an Automated Analysis. *Microbes Environ ***41**: ME25070.

https://doi.org/10.1264/jsme2.ME25070

## Supplementary Material

Supplementary Material

## Figures and Tables

**Fig. 1.**
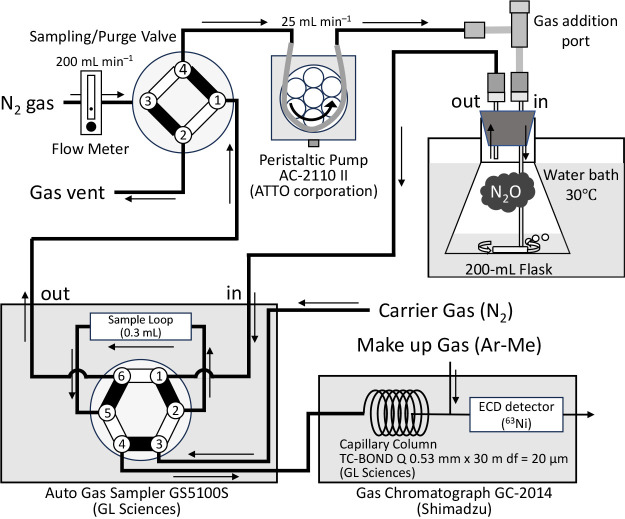
Diagram of the automated continuous N_2_O measurement system. The system consists of a sample flask, a peristaltic pump for gas circulation, a sample loop, and a gas chromatograph (GC) equipped with an electron capture detector (ECD). The diagram shows the gas flow path (indicated by arrows) from the headspace of the flask through the sample loop for repeated sampling and injection into the GC, which allows for the real-time monitoring of N_2_O concentrations. To maintain anaerobic conditions, the system uses a Sampling/Purge Valve (a 4-port, 2-position valve): for sampling through the sample loop, the black lines are used (1–4, 3–2); and for purging the system with N_2_ gas, the white lines are used (1–2, 3–4). Additionally, the internal valve position of the Auto Gas Sampler changes depending on the measurement cycle: during the “Injection” step, the valve is set to the white lines (1–6, 5–4, 3–2) to inject the sample loop gas into the GC; otherwise, the black lines (1–2, 3–4, 5–6) are used for continuous circulation.

**Fig. 2.**
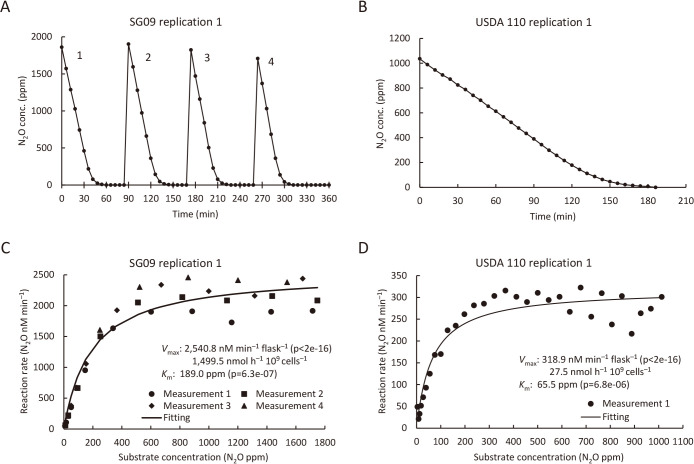
Kinetic anal­ysis of N_2_O reduction by *Bradyrhizobium ottawaense* SG09 and *B. diazoefficiens* USDA110. (A, B) Changes in N_2_O consumption over time by (A) SG09 and (B) USDA110, as measured by gas chromatography. (C, D) Michaelis–Menten plots showing the relationship between N_2_O concentrations and the reaction rates of (C) SG09 and (D) USDA110. Panels show representative results from one of three independent replicate experiments. Data from all three replicates are available in the Supplementary Information as Fig. S5 and Fig. S6.

**Table 1.** Estimated *K*_m_ and *V*_max_ of N_2_O-reducing activities of *Bradyrhizobium ottawaense* SG09 and *B. diazoefficiens* USDA110

Strain	Replication	Cell number cells flask^–1^	*K*_m_ ppm N_2_O	*V*_max_		*V*_340 ppb N2O_ nmol N_2_O h^–1^ 10^9^ cells^–-1^
				nM N_2_O min^–1^ flask^–1^	nmol N_2_O h^–1^ 10^9^ cells^–1^	
SG09	Rep_1	2.3.E+10	189.0 (6.3e-07)	2540.8 (<2e-16)	1499.5	
	Rep_2	2.5.E+10	265.3 (1.6e-09)	2657.7 (<2e-16)	1414.0	
	Rep_3	2.4.E+10	228.7 (3.7e-09)	2610.1 (<2e-16)	1499.4	
	Average		227.7±38.2		1470.9±49.4	2.24±0.44
USDA110	Rep_1	1.0.E+11	65.5 (6.8e-06)	318.9 (<2e-16)	27.5	
	Rep_2	9.5.E+10	68.0 (6.1e-03)	223.6 (<2e-16)	24.0	
	Rep_3	8.4.E+10	70.9 (1.8e-05)	202.1 (<2e-16)	19.2	
	Average		68.1±2.7		23.6±4.1	0.12±0.03

Kinetic parameters were assessed by fitting N_2_O reduction data to the Michaelis–Menten equation. Values represent the means±standard deviation of three independent replicate experiments. Values in parentheses are *P*-values for the Michaelis–Menten equation fit.

**Table 2.** Estimated kinetic parameters of N_2_O-reducing activities of SG09, USDA110, and previously reported clade I and II *nosZ*-carrying bacteria

Strain	*nosZ* type	*K*_m_ (μM)	*V*_max_ (mol g DW^–1^ h^–1^)	Reference
*Bradyrhizobium ottawaense* SG09	Clade I	9.16	0.007	This study
*Bradyrhizobium diazoefficiens* USDA110	Clade I	2.72	0.0003	This study
*Pseudomonas stutzeri* JCM5965	Clade I	4.01	0.008	Suenaga *et al.*, 2018
*Pseudomonas stutzeri* DCP-1	Clade I	35.5	0.250	Yoon *et al.*, 2016
*Shewanella loihica* PV-4	Clade I	7.07	0.027	Yoon *et al.*, 2016
*Paracoccus denitrificans* NBRC102528	Clade I	34.8	0.003	Suenaga *et al.*, 2018
*Cloacibacterium* sp. CB-01	Clade II	12.9	0.006	Hiis *et al.*, 2024
*Dechloromonas aromatica* RCB	Clade II	0.32	0.028	Yoon *et al.*, 2016
*Anaeromyxobacter dehalogenans* 2CP-C	Clade II	1.34	0.001	Yoon *et al.*, 2016
*Azospira* sp. I09	Clade II	1.55	0.021	Suenaga *et al.*, 2019
*Azospira* sp. I13	Clade II	2.10	0.090	Suenaga *et al.*, 2019

*V*_max_ values cell^–1^ dry weight of SG09 and USDA110 were calculated using the methodology of Hiis *et al.* (2024). The *K*_m_ and *V*_max_ values for other bacteria were cited from the study by Hiis *et al.* (2024) and its references.
