# Supplementary material for: Kinetics of Nitrous Oxide (N2O)-reducing Activity of Bradyrhizobium ottawaense by an Automated Analysis
Source: Microbes Environ. 2026 Jan 28;41(1):ME25070. doi: 10.1264/jsme2.ME25070 (PMC12999729; doi:10.1264/jsme2.ME25070)
Supplement: Supplementary file 1 — Supplementary Material [file 41_25070_s1.pdf]

A: Atmospheric condition

B: Anaerobic 1,400 ppm N_2O condition

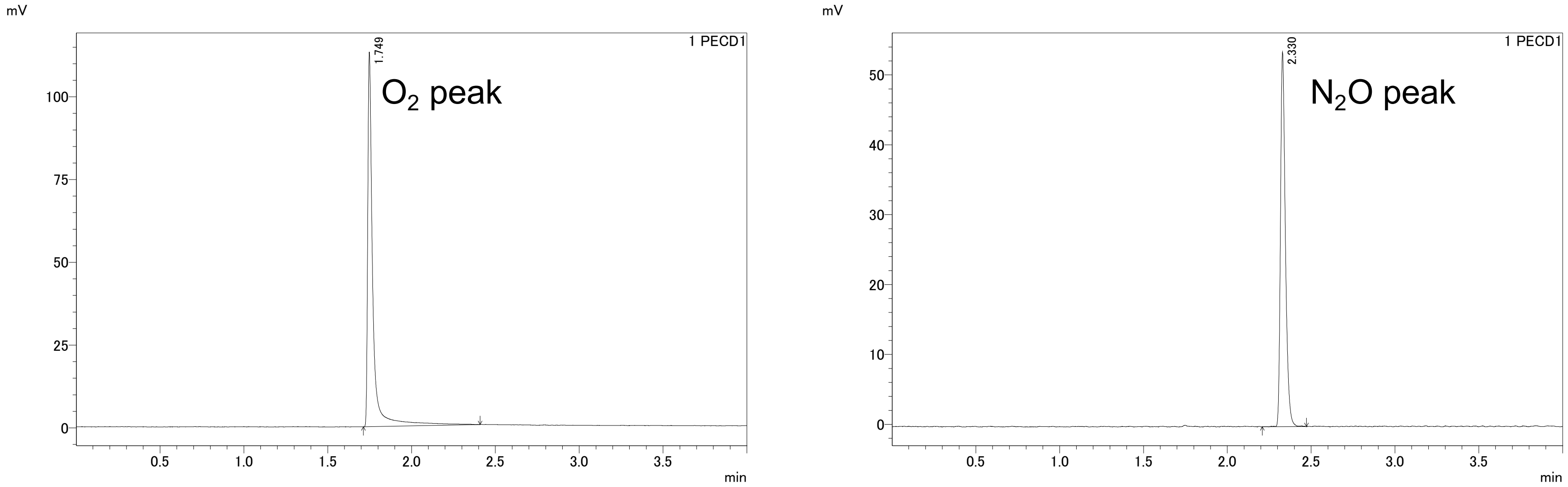

Fig. S1. Representative gas chromatograms from the Electron Capture Detector (ECD) for method validation. Panel A shows the gas profile under an atmospheric condition, and Panel B shows the profile under the anaerobic, N_2O-added condition used for kinetic analysis.

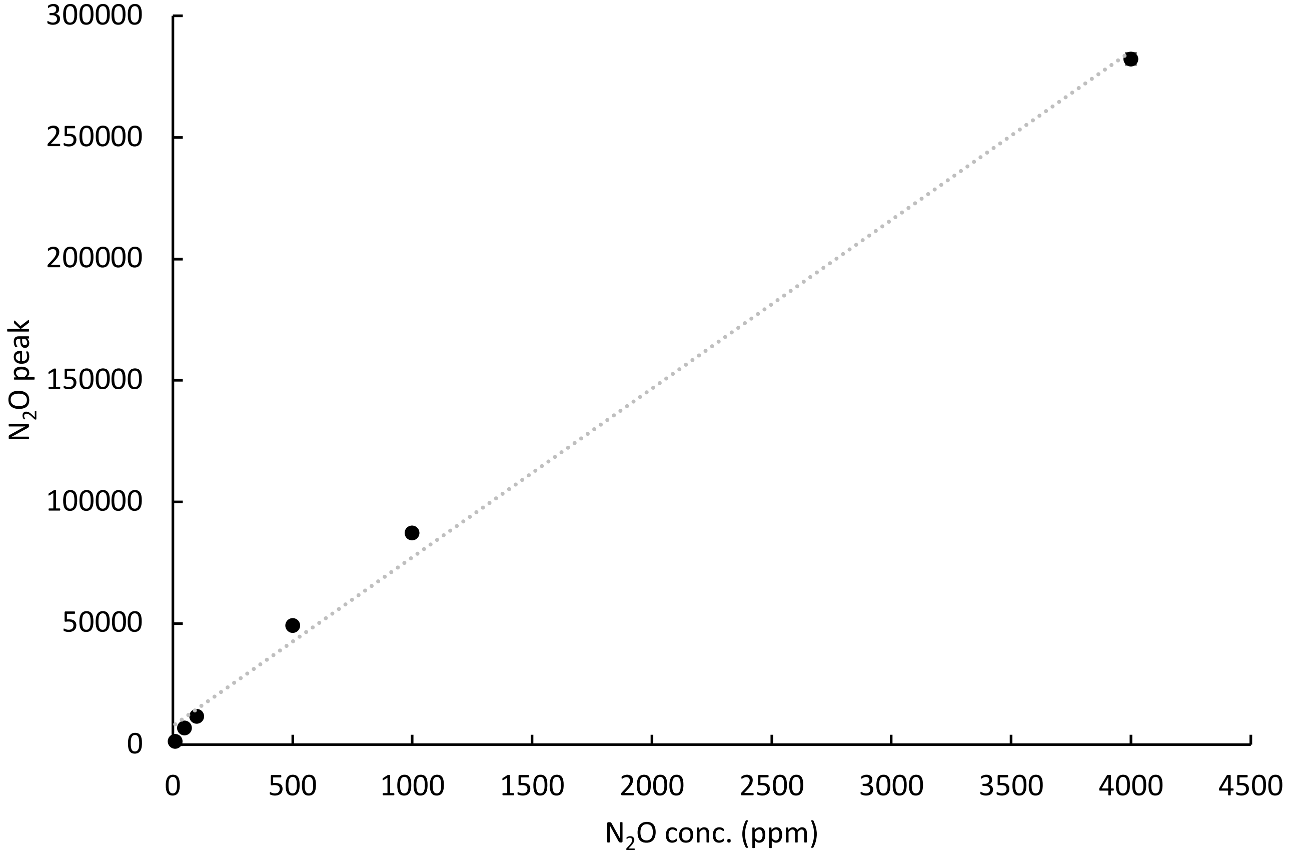

Fig. S2. Calibration curve for the automated N_2O measurement system.
The relationship between N_2O concentration of standard gases (10, 50, 100, 500, 1000, and 4000 ppm in N_2) and the corresponding peak area measured by the gas chromatograph was analyzed. Measured values are represented by black dots, and the linear approximation is shown as a gray dashed line (Linear Regression, R^2 = 0.996, n = 5).

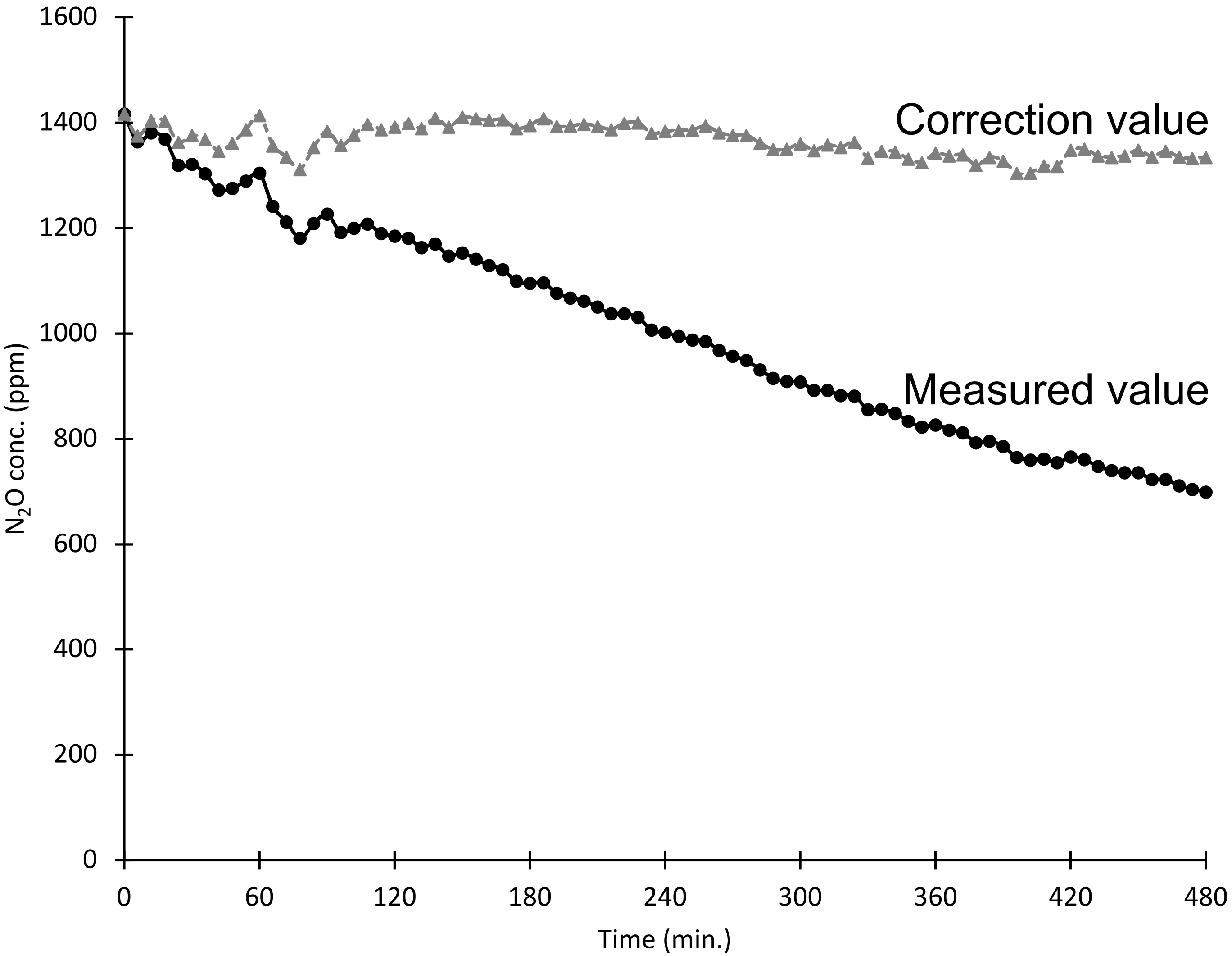

Fig. S3. Verification of the automated continuous N_2O measurement system. Changes in N_2O concentration over time in the absence of microorganisms demonstrate the stability and reliability of the measurement system. Both the raw measured values and the corrected values, accounting for the 0.3 mL nitrogen gas influx into the headspace during each measurement, are shown.

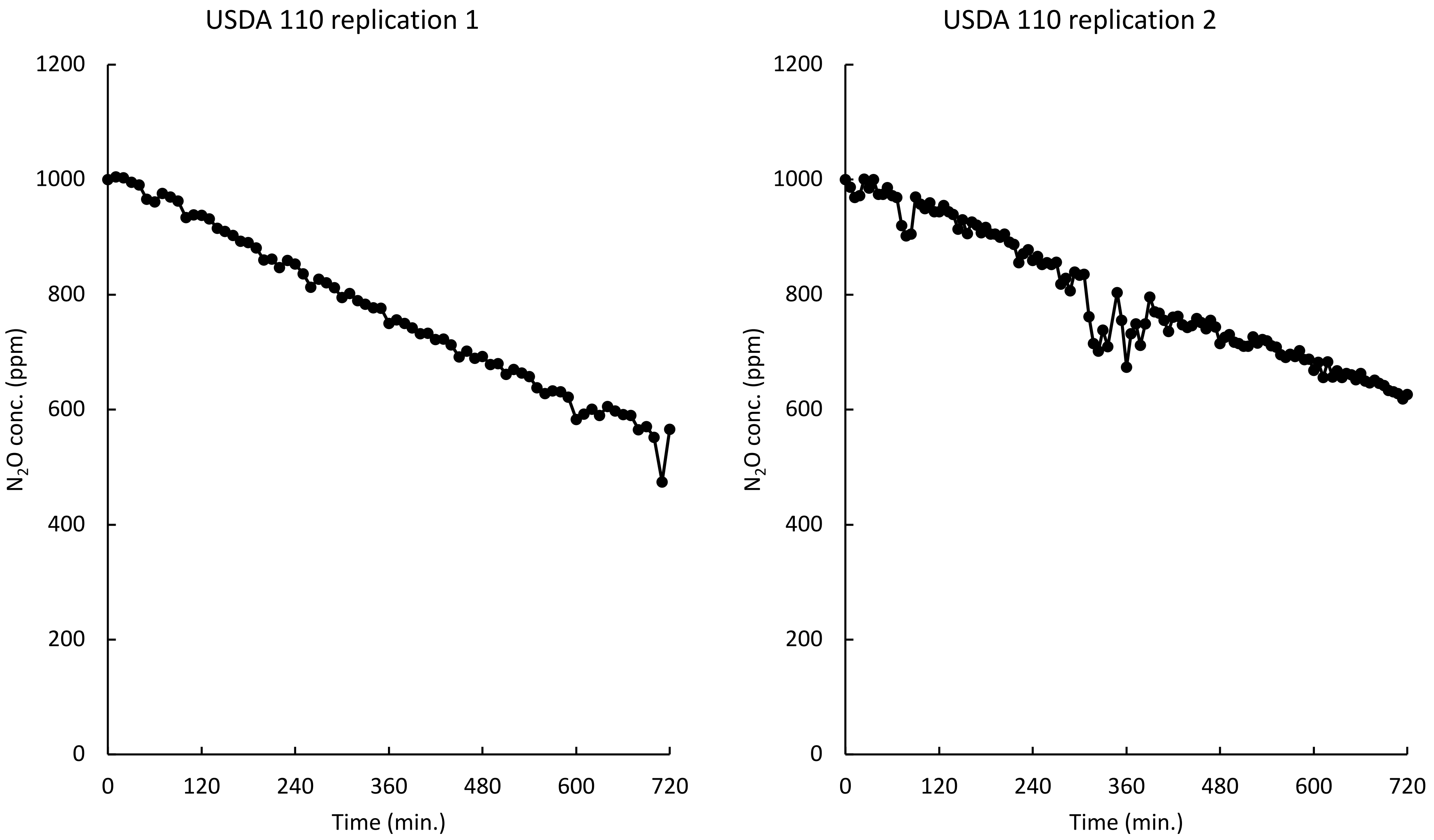

Fig. S4. Kinetic analysis of N_2O reduction activity in *B. diazoefficiens* USDA 110 under the same experimental conditions (cell density and suspension volume) as *B. ottawaense* SG09.

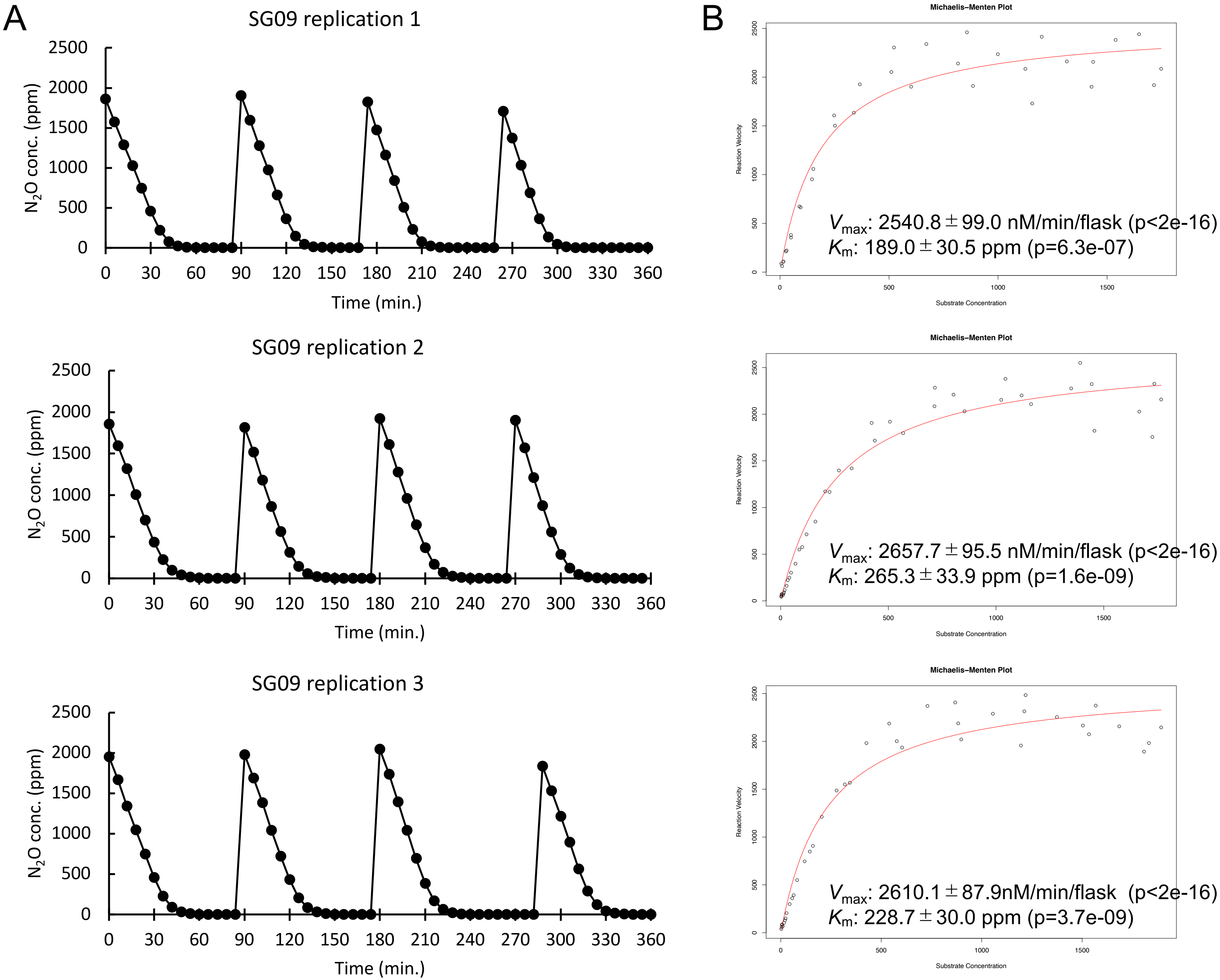

Fig. S5. Kinetics analysis of N_2O reduction in *B. ottawaense* SG09. (A) Changes in N_2O consumption over time measured by gas chromatography. (B) Fitting to the Michaelis-Menten equation by R version 4.2.2.

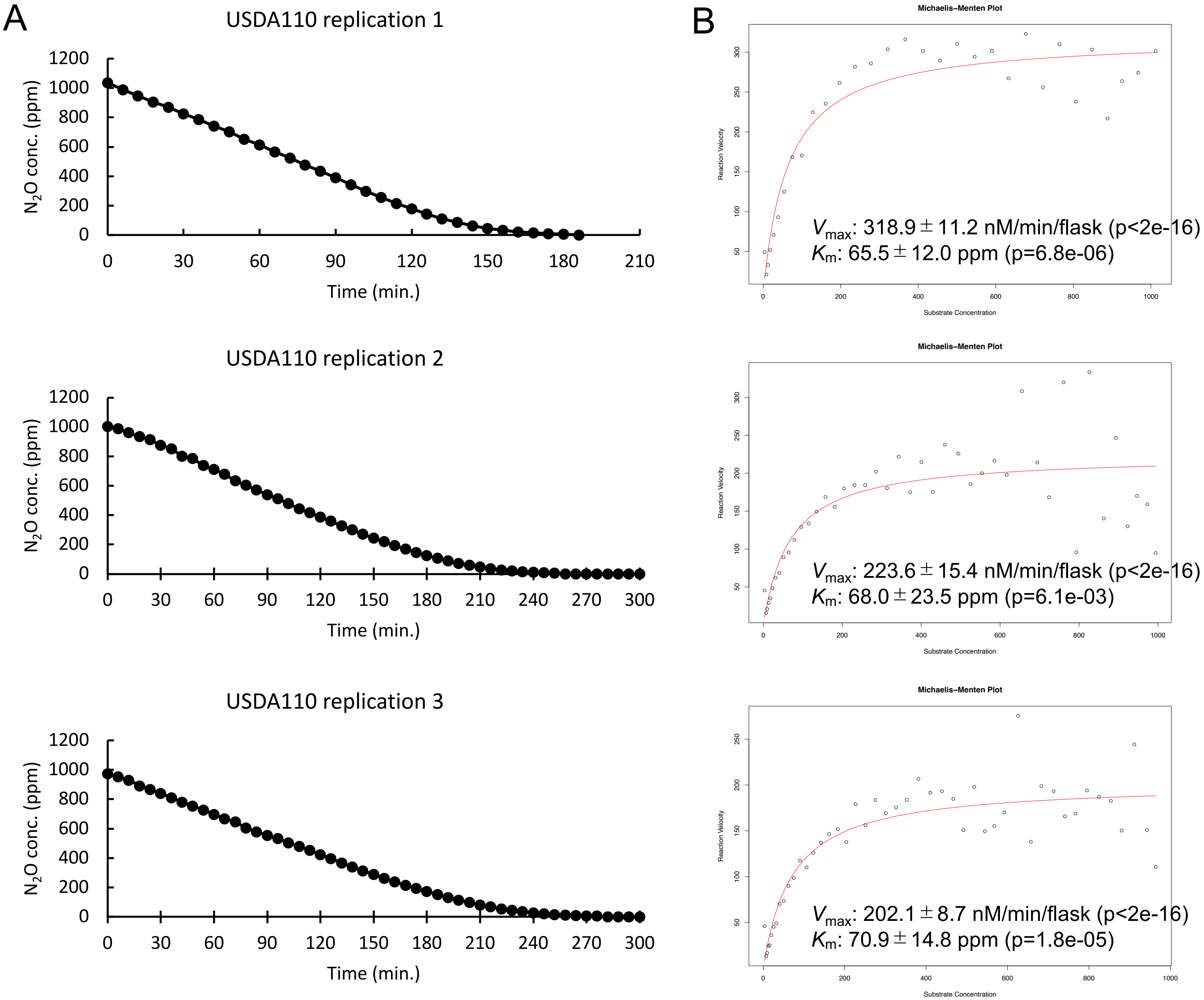

Fig. S6. Kinetics analysis of N_2O reduction in *B. diazoefficiens* USDA 110. (A) Changes in N_2O consumption over time measured by gas chromatography. (B) Fitting to the Michaelis-Menten equation by R version 4.2.2.
